# Phylogenetic inference reveals clonal heterogeneity in circulating tumor cell clusters

**DOI:** 10.1038/s41588-025-02205-2

**Published:** 2025-06-02

**Authors:** David Gremmelspacher, Johannes Gawron, Barbara M. Szczerba, Katharina Jahn, Francesc Castro-Giner, Jack Kuipers, Jochen Singer, Francesco Marass, Ana Gvozdenovic, Selina Budinjas, Heike Pueschel, Cyrill A. Rentsch, Alfred Zippelius, Viola Heinzelmann-Schwarz, Christian Kurzeder, Walter Paul Weber, Christoph Rochlitz, Marcus Vetter, Niko Beerenwinkel, Nicola Aceto

**Affiliations:** 1 Department of Biology, Institute of Molecular Health Sciences, ETH Zurich, Zurich, Switzerland; 2 Department of Biosystems Science and Engineering, ETH Zurich, Basel, Switzerland; 3 SIB Swiss Institute of Bioinformatics, Basel, Switzerland; 4 Department of Biomedicine, University of Basel, Basel, Switzerland; 5 Department of Mathematics and Computer Science, Freie Universität Berlin, Berlin, Germany; 6 Department of Urology, University Hospital Basel and University of Basel, Basel, Switzerland; 7 Gynecologic Cancer Center, University Hospital Basel, Basel, Switzerland; 8 Breast Center, University of Basel and University Hospital Basel, Basel, Switzerland; 9 Department of Medical Oncology, University Hospital Basel, Basel, Switzerland; 10 Cancer Center Baselland Medical University Clinic, Kantonsspital Baselland, Liestal, Switzerland

**Keywords:** Breast cancer, Genome informatics

## Abstract

Circulating tumor cell (CTC) clusters are highly efficient metastatic seeds in various cancers. Yet, their genetic heterogeneity and clonal architecture is poorly characterized. Using whole-exome sequencing coupled with phylogenetic inference from CTC clusters of patients with breast and prostate cancer, as well as mouse cancer models alongside barcode-mediated clonal tracking in vivo, we demonstrate oligoclonal composition of individual CTC clusters. These results improve our understanding of metastasis-relevant clonal dynamics.

## Abstract (web summary)

Phylogenetic analyses in patients with cancer and xenograft models highlight heterogeneity within individual CTC clusters, providing insights into clonal dynamics during metastasis.

## Main

**Fig. 1:**
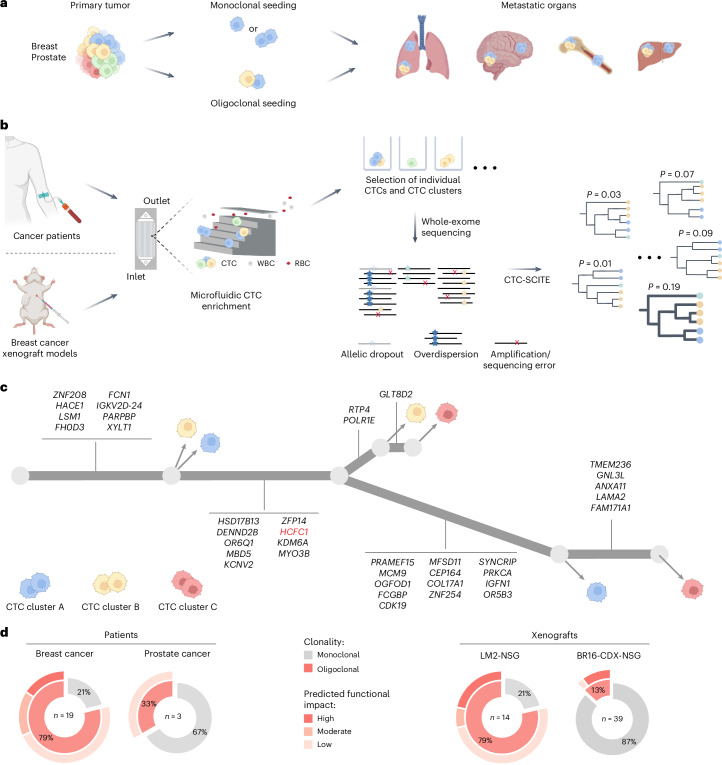
Phylogenetic inference reveals CTC cluster oligoclonality in carcinoma patient samples and breast cancer xenografts. **a**, Schematic representation of clonal architectures of CTCs during cancer metastasis. **b**, Experimental and computational strategy for deriving phylogenetic trees from CTC mutational profiling. RBC, red blood cell. **c**, Best-fitting phylogenetic tree (simplified) for patient with breast cancer ‘Br61’ obtained with CTC-SCITE, highlighting three CTC clusters inferred as oligoclonal after statistical evaluation of the probability of branching evolution among their constituent cells. Cell colors reflect CTC cluster identity. Genes with moderate or high predicted functional impact on protein activity are depicted. Oncogenic drivers predicted by the Cancer Genome Interpreter are highlighted in red. Panels **a**–**c** are created with BioRender.com. **d**, Proportion of monoclonal and oligoclonal CTC clusters (inner circle) inferred for patient samples (left) and breast cancer xenograft samples (right). For oligoclonal CTC clusters, the fraction of CTC clusters with low, moderate and high predicted functional impact of lineage-defining mutations is depicted (outer circle). The total number of examined CTC clusters (*n*) for each cancer type and xenograft model is provided. Source data

Genetic intratumor heterogeneity is a hallmark of cancer and is associated with poor prognosis in patients with cancer^1–3^. Acquisition of multiple, diverse genomic alterations in different malignant cells over time results in the emergence of genetically divergent tumor subclones, evolving through genetic drift, natural selection and cell dispersal^4^. Circulating tumor cells (CTCs) are shed from solid tumors as precursors of metastasis and reflect the genetic profile of their clone of origin. Therefore, DNA sequencing of CTCs can be regarded as a minimally invasive approach to infer clonal composition of CTC-shedding areas of both primary tumors and metastases. CTCs travel through the bloodstream as individual cells or as multicellular aggregates known as CTC clusters^5–8^. Increasing evidence links intratumor heterogeneity to oligoclonal metastatic seeding^9–12^, a consequence of either sequential homing of CTCs originating from distinct tumor clones or seeding of oligoclonal CTC clusters (Fig. 1a). CTC clusters have increased metastatic capacity compared to single CTCs in vivo^8,13^, and their detection in patients with cancer is associated with worse clinical outcomes across multiple cancer types^14–16^, highlighting their potential key role in metastasis development. Therapies to disrupt CTC clusters are emerging, such as inhibitors of the Na^+^K^+^ ATPase, recently showing proof-of-mechanism cluster dissolution in the clinic^17^. As the spread of cancer accounts for the vast majority of cancer-related deaths, a better understanding of the clonal dynamics underlying this phenomenon is required.

The clonal diversity of cells within individual CTC clusters in human malignancies is poorly defined. Prior investigations conducted in breast cancer mouse models suggested oligoclonality in CTC clusters^8,18^. Yet, these studies inferred clonality from the presence of different ectopic optical labels rather than actual genetic divergence of cells within CTC clusters, thus reflecting genetic heterogeneity of tumors only in part. Clinical data providing substantial evidence for (or against) the presence and prevalence of oligoclonal CTC clusters in human cancer are lacking.

**Extended Data Fig. 1:**
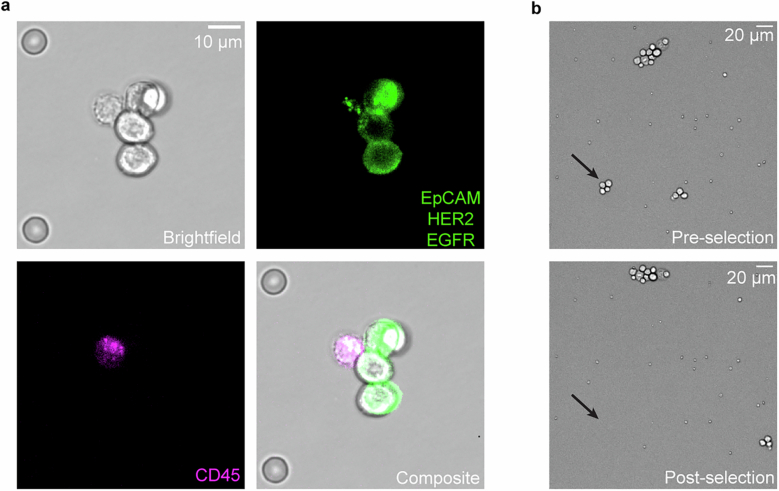
Patient-derived and xenograft-derived CTC clusters. **a**, Representative microscopic images of patient-derived CTC clusters stained for EpCAM, HER2 and EGFR (CTCs, green) and CD45 (white blood cells, magenta). Pseudo-coloring and gamma adjustments were applied to fluorescent images. Scale bar, 10 µm. **b**, Representative images of xenograft-derived CTC clusters preselection and postselection via robotic micromanipulation. The black arrow points to the targeted CTC cluster. Scale bar, 20 µm.

Here we conducted a proof-of-principle study to interrogate the clonality of CTC clusters in the blood of patients with cancer and xenograft mouse models directly by using customized phylogenetic methods. We enriched CTCs from peripheral blood samples of patients with progressing metastatic breast and prostate carcinoma (Supplementary Table 1), as well as from two different breast cancer xenograft models using the FDA-approved microfluidic platform Parsortix (Fig. 1b). The patient study cohort comprised seven patients with breast cancer, enrolled in a previous CTC study^13^, as well as two patients with prostate cancer with positive CTC cluster count. Mouse models included NOD.Cg-*Prkdc*^scid^-*Il2rg*^tm1Wjl^/SzJ (NSG) xenografts, obtained by mammary fat pad injection of green fluorescent protein (GFP)-labeled human breast CTCs (BR16-CDX-NSG) or established human MDA-MB-231 LM2 breast cancer cells (LM2-NSG). We identified CTCs based on staining for cancer-associated cell surface markers EpCAM, HER2 and EGFR (patient samples) or upon detection of GFP signal (xenograft models). CD45 staining was used to identify white blood cells (WBCs; Extended Data Fig. 1a). Upon capture, we harvested CTCs and CTC clusters from patient and xenograft samples using robotic micromanipulation (Extended Data Fig. 1b), transferring cells into individual tubes for genomic analysis. Whenever feasible, we physically dissociated CTC clusters into individual cells through gentle micromanipulation and collected cells in separate tubes. All samples (either single cells or inseparable CTC clusters) were then subjected to whole-exome sequencing to obtain read count profiles.

**Extended Data Fig. 2:**
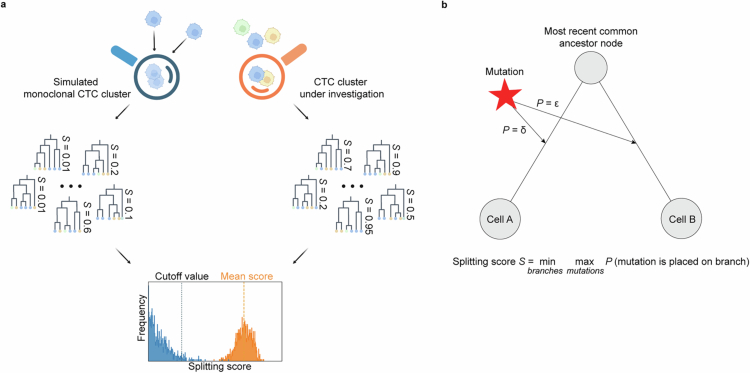
Assessment of clonality in CTC clusters. **a**, Schematic representation of the strategy used to estimate the clonality of patient-derived and xenograft-derived CTC clusters. For each patient and xenograft, genealogical tree topologies are sampled from the posterior distribution using CTC-SCITE. For each CTC cluster, we determine the distribution of splitting scores (*S*), reflecting the probability of branching (versus linear) evolution between pairs of cells (orange histogram). In parallel, monoclonal CTC clusters matching the genotypes of single CTCs are simulated to provide an estimate of the distribution of splitting scores in monoclonal CTC clusters (null distribution; blue histogram). A CTC cluster is assigned oligoclonal if the mean splitting score (dashed orange vertical line) exceeds the 95-percentile of the null distribution (dotted blue vertical line). Panel **a** is created with BioRender.com. **b**, Schematic representation of the inference of splitting scores (*S*) for pairs of individual CTC cluster-derived cells. For a selected pair of cells within a given phylogeny sampled from the posterior distribution of tree topologies, we inspect the paths to their most recent common ancestor. A CTC cluster splits with high confidence if at least one mutation is mapped with high probability to each of the two branches, and it splits with low confidence if all mutations are mapped with low probability to one or both branches. We account for this by computing each mutation’s probability (*P*) of mapping to either of the two branches A (*P* = *δ*) and B (*P* = *ε*) and consider the maximum probability of any mutation mapping to it. The splitting score *S* reflects the lower of those two maximal probabilities.

We developed a Bayesian phylogenetic tree inference model (CTC-SCITE; for a comprehensive description of the model, refer to Supplementary Note 1) based on the algorithms SCITE^19^ and SCIΦ^20^ to infer the genealogy of the sequenced single cells from their mutation profiles and the clonality of CTC clusters from the genealogical relationships of the constituent cells. Our model is able to deconvolve the aggregate read count profiles of CTC clusters (Supplementary Note 1), placing constituent cells in the most likely configuration on the phylogenetic tree (Fig. 1c) and inferring their genotypes even when they could not be physically dissociated into single cells. To determine the clonality of CTC clusters, we sampled trees from their posterior distribution and assessed the probability of branching evolution for pairs of individual CTC cluster-derived cells. We statistically evaluated whether the observed probabilities significantly deviated from the null distribution for pairs of genetically convergent cells in simulated monoclonal CTC clusters (Extended Data Fig. 2a,b) and inferred an oligoclonal composition of CTC clusters whenever the null hypothesis of no branching evolution among cells was rejected. We further determined mutations that were exclusive to specific cells within an oligoclonal CTC cluster. When this exclusivity pattern was consistent throughout sampled trees, we identified these mutations as markers of the genetically distinct lineages. We categorized oligoclonal CTC clusters based on the predicted functional impact of these lineage-defining mutations on protein activity from low (unlikely to change protein function) to high (strong disruptive impact, for example, causing protein truncation, loss of function or nonsense mediated decay) and further annotated lineage-defining alterations based on their putative oncogenic impact.

We found evidence for branching evolution in 16 of 22 (73%) patient-derived CTC clusters, including 15 breast and one prostate cancer-derived CTC cluster (Fig. 1d). Among the 16 CTC clusters with branching evolution, we observed moderate to high functional impact of lineage-defining mutations in 6 of 15 (40%) and 0 of 1 (0%) CTC clusters of patients with breast and prostate cancer, respectively. For the breast cancer xenograft-derived CTCs, we found branching evolution in 11 of 14 (79%) CTC clusters from the fast-growing LM2-NSG model and in 5 of 39 (13%) CTC clusters from the slow-growing BR16-CDX-NSG model. Lineage-defining mutations with relevance on protein activity were identified in 4 of 11 (36%) and 4 of 5 (80%) CTC clusters with branching evolution in the LM2-NSG and BR16-CDX-NSG model, respectively. Altogether, phylogenetic inference provides evidence of genetic heterogeneity in CTC clusters of patients with breast and prostate cancer, as well as breast cancer xenograft models.

**Fig. 2:**
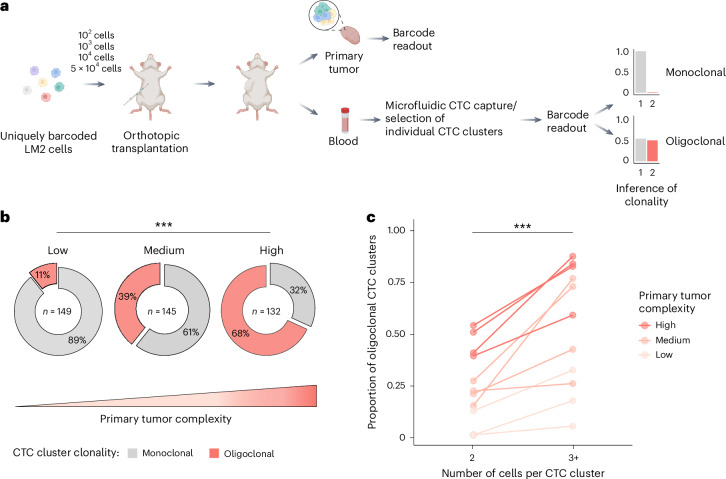
CTC cluster clonality is associated with primary tumor clonal complexity and CTC cluster size. **a**, Schematic representation of the experimental strategy used to model clonal expansion with varying clonal complexities and infer the prevalence of oligoclonal CTC clusters. Panel **a** is created with BioRender.com. **b**, Proportion of oligoclonal CTC clusters disseminated from tumors with low, medium and high clonal complexities (***P < 1 × 10^−15^, Cochran–Armitage test, *Z* = 9.89). The total number of interrogated CTC clusters (*n*) is specified for each primary tumor complexity. **c**, Proportion of oligoclonality in CTC clusters with two cells and three or more cells (***P = 3.7 × 10^−7^, Fisher’s exact test (two-sided), odds ratio = 0.33, 95% confidence interval = 0.20–0.52). All mouse samples with detection of CTC clusters in both categories are shown (*n* = 3 for low, *n* = 4 for medium and *n* = 4 for high primary tumor complexities). Source data

**Extended Data Fig. 3:**
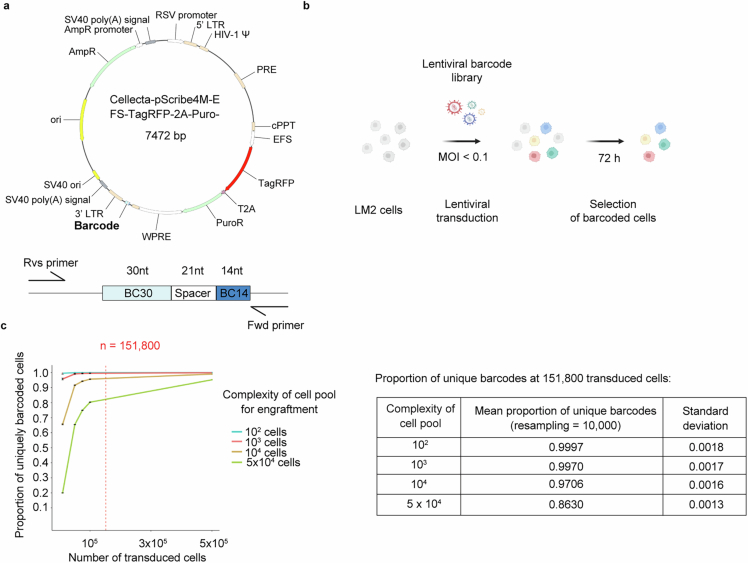
Molecular barcoding of LM2 cells. **a**, Clonetracker XP plasmid map and structure of the barcode cassette obtained from SnapGene software v7.0.0 (from Dotmatics; available at snapgene.com). **b**, Graphical representation of the experimental design with target multiplicity of infection. **c***,* Left, plot showing the simulated mean proportion of uniquely barcoded cells within sampled cell pools of varying complexities for in vivo engraftment as a function of the number of initially transduced cells (10,000× resampling; Supplementary Note 2). Error bars, s.d. Vertical red line at 151,800 represents the actual number of transduced cells in the conducted experiment as determined via FACS. Right, table depicting the mean and s.d. of the proportion of unique barcodes in sampled pools of 10^2^, 10^3^, 10^4^ and 5 × 10^4^ cells, 72 h after successful transduction of 151,800 cells (10,000× resampling). Panel **b** is created with BioRender.com. MOI, multiplicity of infection.

**Extended Data Fig. 4:**
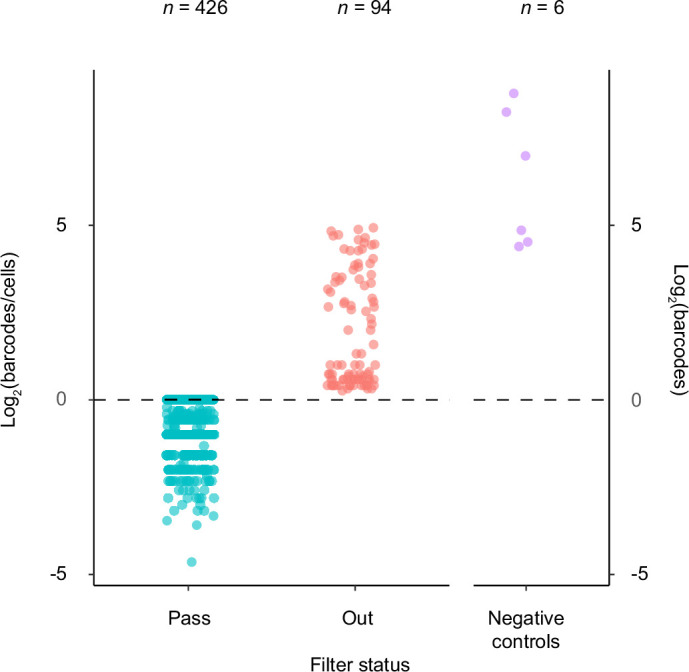
Quality filtering of barcoded CTC clusters. Plot showing the ratio of the smallest number of barcodes accumulating 90% of total aligned reads over the number of cells per cluster (*y* axis, log_2_ scale), for CTC clusters included (number of barcodes/number of cells > 1, filter status = pass, *n* = 426) and removed (number of barcodes/number of cells > 1, filter status = out, *n* = 94) from the analysis. The horizontal line at *y* = 0 illustrates the cut-off for filtering. For comparison, the smallest number of barcodes accumulating 90% of aligned reads in negative control samples (*n* = 6), containing only lysis buffer without cells, is depicted.

**Extended Data Fig. 5:**
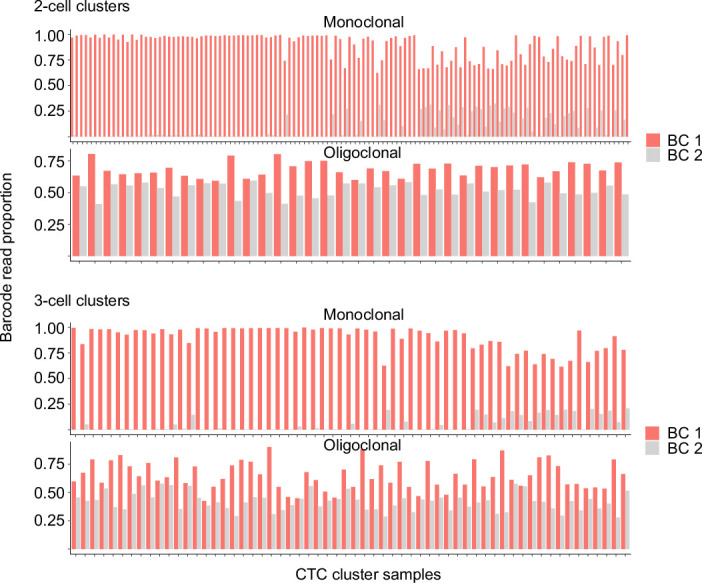
Calling clonality in barcoded CTC clusters. Plots for two-cell CTC clusters (top) and three-cell CTC clusters (bottom) show, for each CTC cluster labeled monoclonal (BC#1 > BC#2 × number of cells, top) or oligoclonal (BC#1 ≤ BC#2 × number of cells, bottom), the fraction of total aligned reads for the most dominant (BC#1, red) and the second most dominant (BC#2, gray) barcode. BC, barcode.

**Extended Data Fig. 6:**
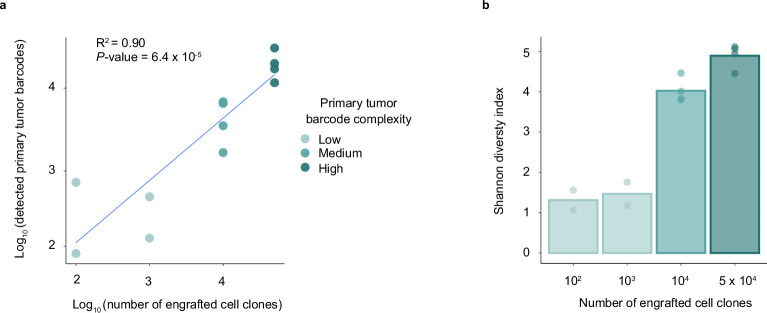
Correlation of detected primary tumor barcodes with engrafted cell counts, and Shannon diversity index of barcode populations. **a**, Number of detected barcodes (*y* axis, log_10_ scale, cutoff of ten counts per million) as a function of the number of engrafted barcoded cell clones (*x* axis, log_10_ scale) in sequenced primary tumor samples (Pearson´s correlation coefficient *R*^2^ = 0.90, *P* = 6.4 × 10^−5^, 95% confidence interval = 0.68–0.97, two-sided). Points are colored according to classification into low, medium and high complexity based on the Shannon diversity index. **b**, Shannon diversity index for clonal barcode populations in tumors grown from 10^2^ (*n* = 2), 10^3^ (*n* = 2), 10^4^ (*n* = 4) and 5 × 10^4^ (*n* = 4) engrafted barcoded cell clones. Bars depict the mean.

**Extended Data Fig. 7:**
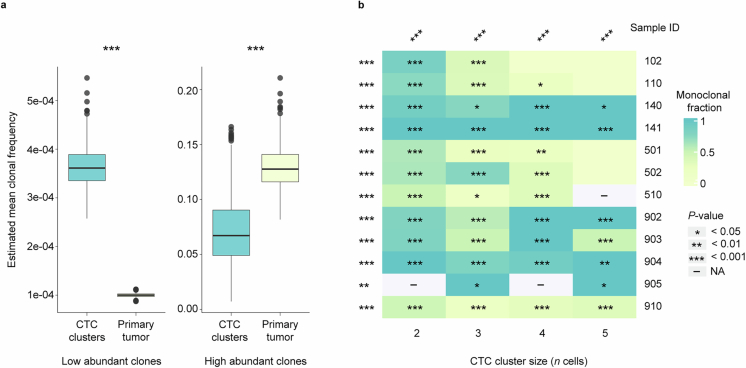
Comparing primary tumor clonal frequencies with clonal prevalence in CTC clusters, evaluating the expected versus observed fraction of oligoclonal CTC clusters. **a**, Plot showing the mean clonal frequencies of barcodes in primary tumor and CTC clusters stratified by primary tumor abundance (‘high’ for clones within the 99.9 percentile of the empirical distribution of its relative abundances in primary tumors, and ‘low’ for clones outside the 99.9 percentile). Bootstrapping (sample size = 1,000) addresses uncertainty in the estimate. The centers of the boxplots are defined as the medians of the estimates, top and bottom hinges show the first and third quartiles, respectively, and whiskers reach out to the furthest points whose distance from the hinges is smaller than 1.5 times the interquartile range. All outliers are plotted as points. The distortion of clonal representation in CTC clusters is significant (combined one-sided, ***P < 1 × 10^−15^, χ^2^ = 3,992.58 with 852 degrees of freedom; Supplementary Note 3). **b**, Heatmap illustrating the inferred fraction of monoclonality among CTC clusters, stratified by CTC cluster size (*x* axis, depicted are CTC clusters with two to five cells) and mouse sample (*y* axis). *P* values (one-sided) represent significance levels for the deviation from expected monoclonality levels by random clonal mixing (Supplementary Note 4). *P* values at the margins represent the combined *P* values obtained by Fisher’s method. NA, not applicable.

We next sought to assess the prevalence of oligoclonal CTC clusters as a function of the clonal diversity of primary tumors. We reasoned that the clonality of CTC clusters could reflect the clonal composition at the intravasation sites and hypothesized that the prevalence of oligoclonal CTC clusters would increase along with the clonal diversity of originating primary tumors. CTC clusters are rare in peripheral blood samples of patients with cancer, and matched primary tumor information reflective of intratumor heterogeneity is typically lacking due to tissue sampling constraints. Therefore, we modeled clonal expansion in tumors of varying clonal configurations using an orthotopic xenograft mouse model with clonally labeled breast cancer cells. We labeled LM2 human breast cancer cells with molecular barcodes using a high-complexity lentiviral library consisting of 4.8 million unique barcodes (Extended Data Fig. 3a,b), aiming to obtain pools of uniquely barcoded cells for in vivo transplantations (Extended Data Fig. 3c and Supplementary Note 2). Our experimental design allowed engraftment of increasing numbers of barcoded LM2 cells into the mammary fat pad of female NSG mice, resulting in orthotopic breast cancer lesions of varying clonal barcode complexities. Upon reaching the final stage of tumor growth, terminal blood sampling and microfluidic CTC capture were performed, followed by micromanipulation and deep targeted sequencing, enabling barcode readouts in individual CTC clusters to infer clonality (Fig. 2a). In total, after quality filtering (Extended Data Fig. 4), 426 CTC clusters were included in the analysis (Supplementary Table 2) and determined monoclonal (one dominant barcode) or oligoclonal (two or more dominant barcodes; Extended Data Fig. 5). In parallel, to determine clonal diversity of CTC-generating tumors, these were resected and subjected to targeted barcode sequencing. In accordance with findings from previous lineage tracing experiments^21,22^, we observed a strong clonal drop-out following orthotopic transplantation. Nonetheless, primary tumor clonal composition accurately recapitulated the variations in clonal complexity (or simply, number of cells) present in the original cell pools before orthotopic transplantation (Extended Data Fig. 6a). Using the Shannon diversity index to quantify clonal diversity, we found tumor diversity to increase with the number of engrafted cell clones, although the difference was not statistically significant between 10^2^ and 10^3^ engrafted cells (Extended Data Fig. 6b). Consequently, these tumors were classified together as low-barcode-complexity tumors, while tumors with 10^4^ and 5 × 10^4^ injected cell clones were assigned as medium-barcode-complexity and high-barcode-complexity tumors, respectively. Interestingly, we found that dominant primary tumor clones were represented in CTC clusters at lower levels than expected from their clonal frequencies in the primary tumor (Extended Data Fig. 7a), suggesting that clonal frequencies in the primary tumor are not the only determinant of clonal prevalence at the level of CTC clusters (*P* < 1 × 10^−15^; Supplementary Note 3). We speculate that other factors could be at play in this context, including cancer cell-intrinsic features and local microenvironmental signals, both likely to influence the dynamics of CTC cluster formation. The observed overall proportion of oligoclonal CTC clusters across all cluster sizes and primary tumor diversities was 38%, increasing from 11% for low-complexity tumors to 39% for medium-complexity tumors and 68% for high-complexity tumors (Fig. 2b) and indicating a strong association between primary tumor clonal complexity and the probability of oligoclonal CTC cluster formation (*P* < 1 × 10^−15^, Cochran–Armitage test). Of note, CTC clusters were found to be monoclonal more frequently than expected when randomly selecting clones from the primary tumor (Extended Data Fig. 7b and Supplementary Note 4). Finally, we aimed to evaluate how the size of CTC clusters (that is, the number of cells per cluster) contributed to their clonality. We observed an increase in the proportion of oligoclonal clusters as the number of cells per CTC cluster increased from two to three or more cells across low-complexity, medium-complexity and high-complexity tumors (*P* = 3.7 × 10^−7^, Fisher’s exact test; Fig. 2c), providing evidence for a higher likelihood of oligoclonality as a function of cluster size.

In summary, our findings highlight the existence of individual CTC clusters carrying cells from different tumor clones and suggest that quantitative assessment of the clonal composition of CTCs in liquid biopsies could provide insights about the genetic diversity of corresponding primary or metastatic lesions (or at least their CTC-generating portions), potentially mitigating the spatial and temporal limitations of traditional tissue biopsies. Future studies involving large patient cohorts will be required to assess the prognostic relevance of CTC cluster genetic heterogeneity, and orthogonal CTC capture technologies may be considered for cross-validation of present and future findings. Considering the rare nature and relatively smaller size of CTC clusters in peripheral blood compared to more central locations^13,23^, potentially resulting in a lower clonal diversity of CTC clusters, we argue that the implementation of innovative and individualized blood sampling strategies will be key to realizing the full potential of CTC cluster interrogation. These include the careful consideration of blood collection sites tailored to specific cancer entities (that is, collecting blood from the tumor-draining vasculature, when possible, rather than the periphery), time-controlled blood collection^24^ and isolation of CTC clusters from large blood volumes via blood apheresis^25^. Although the augmented metastatic potential of clustered CTCs compared to single CTCs can be attributed to both mechanical and phenotypic properties of CTC clusters^26,27^, we speculate that genetic diversity within CTC clusters may further enhance their metastatic capacity, for instance, by increasing therapy resistance opportunities, evasion from the attack of immune cells, as well as adaptability and survival at the metastatic site. In conclusion, our study provides evidence for genetic heterogeneity in individual CTC clusters of patients with cancer and xenografts, proposing them as contributors of genetic diversity in metastasis and as promising targets when aiming to suppress the spread of genetically divergent secondary tumor lesions.

## Methods

### Inclusion criteria and ethical considerations

Between 7.5 and 15 ml of patient blood in EDTA vacutainers was collected upon written informed patient consent. All specimens were obtained at the University Hospital Basel under ethical approval from the Ethics Committee Northwestern and Central Switzerland (EKNZ), in accordance with the Declaration of Helsinki (protocols EKNZ BASEC 2016-00067, EKNZ 2014-329 and EK 321/10). The patients did not receive any participant compensation. The clinical characteristics of interrogated patients with cancer are included in Supplementary Table 1. All mouse experiments were carried out according to institutional and cantonal guidelines (mouse protocol 33688, approved by the cantonal veterinary office of Zurich).

### Cell culture

MDA-MB-231 lung metastatic variant 2 (LM2) human breast cancer cells (obtained from J. Massagué, Memorial Sloan Kettering Cancer Center) were grown in Dulbecco’s Modified Eagle Medium/Nutrient Mixture F-12 (Gibco, 11330032) supplemented with 10% FBS (Gibco, A5256801) and 1× Antibiotic–Antimycotic (Gibco, 15240062) in a humidified incubator at 37 °C with 20% O_2_ and 5% CO_2_. Human CTC-derived BR16 cells were generated as previously described^28^ from a patient with hormone receptor-positive breast cancer at the University Hospital Basel and propagated as suspension cultures in a humidified incubator at 37 °C with 5% O_2_ and 5% CO_2_. LM2 and BR16 cells were labeled with a GFP-luciferase construct through lentiviral transduction. Cell lines do not belong to the list of commonly misidentified cell lines (International Cell Line Authentication Committee) and were confirmed negative for common contaminating microorganisms, including mycoplasma, by an independent laboratory. Cells were not authenticated as authentication is not applicable for the BR16 and LM2 cell lines.

### Molecular barcoding of LM2 cells

For barcoding experiments, LM2-GFP-luciferase cells were transduced with the CloneTracker XP 5M Barcode-3′ Library in vector pScribe4M-RFP-Puro (Cellecta, BCXP5M3RP-1S-V), containing 4.8 million unique barcode combinations packaged into lentiviral particles. Cells were transduced at a multiplicity of infection below 0.1 to obtain a high proportion of cells with a single, unique barcode integration. Seventy-two hours after lentiviral transduction, barcoded cells were selected based on red fluorescent protein (RFP) signal via fluorescence-activated cell sorting and immediately processed for transplantation into mice.

### Mouse experiments

All mouse experiments were carried out according to institutional and cantonal guidelines (mouse protocol number 33688, approved by the cantonal veterinary office of Zurich). Experimental endpoints specified in our approved license, comprising tumor-related factors, as well as behavioral and appearance-related factors, were closely monitored. The tumor size never exceeded the maximum permitted limit of 2,800 mm^3^. Replacement, reduction and refinement (3R) principles were considered and complied with throughout all experiments. Female NSG mice were purchased from The Jackson Laboratory and kept in pathogen-free conditions in a controlled environment with a room temperature maintained at 22 ± 2 °C and relative humidity at 55 ± 10%, according to institutional guidelines. Animals were kept under a standard 12-h light/12-h dark photoperiod.

Orthotopic breast cancer lesions were generated in eight-week-old to ten-week-old NSG females upon injection of 10^6^ LM2-GFP-luciferase or BR16-GFP-luciferase cells into the mammary fat pad. In both cases, breast cancer cells were inoculated in 100 μl of 50% Cultrex Reduced Growth Factor Basement Membrane Extract, Type 2, PathClear (BME, R&D Biosystems, 3533-010-02) in Dulbecco’s PBS (Gibco, 14190144). Terminal blood draws through cardiac puncture for CTC analysis were performed after four to five weeks for LM2-NSG and five months for Br16-CDX-NSG models.

For barcoding experiments, barcoded LM2-GFP-luciferase cells were inoculated in a 1:1 mix of BME and DPBS at densities corresponding to 10^2^, 10^3^, 10^4^ and 5 × 10^4^ cells in 100 μl. Orthotopic breast cancer lesions of varying barcode complexities were induced upon injection of 100 μl of generated cell suspensions into the mammary fat pad of female NSG mice. All animals were injected and sacrificed synchronically to prevent variability due to circadian fluctuations. No animals or data points were excluded from the analysis.

### CTC capture and immunofluorescence staining

Patient-derived CTCs were captured from unprocessed peripheral blood samples using the FDA-approved microfluidic device Parsortix (ANGLE) equipped with Cell Separation Cassettes (ANGLE, GEN3D6.5). In-cassette staining was performed with antibodies against EpCAM-AF488 (1:50; Cell Signaling Technology, CST5198), HER2-AF488 (1:50; BioLegend, 324410), EGFR-FITC (1:25; GeneTex, GTX11400) and CD45-BV605 (1:25; BioLegend, 304042). Mouse-derived CTCs were captured from 0.8 to 1.2 ml of blood in EDTA tubes (Sarstedt, 41.3395.005) collected through cardiac heart puncture using the Parsortix Cell Separation System as described above and identified based on GFP expression due to stable expression of a GFP-Luciferase reporter. Anti-CD45 staining was carried out to identify CD45-positive cells within the cassette. Microscopic images were processed using the Fiji image processing software (v2.14.0).

For barcoding experiments, CTCs were identified based on the expression of both GFP and RFP, due to stable RFP expression from the integrated barcode cassette. All CTCs were released from Cell Separation Cassettes in reversed flow direction with Dulbecco’s PBS onto ultra-low-attachment plates (Corning, 3471-COR) for downstream procedures.

### Micromanipulation of CTCs and CTC clusters

Whenever possible, CTC clusters for exome sequencing were mechanically dissociated through gentle micromanipulation (CellCelector, ALS). Individual cells from dissociated CTC clusters, intact CTC clusters and single CTCs were aspirated using the automated single-cell picking system CellCelector (ALS) and deposited into individual PCR tubes (Axygen, 21-032-501) containing 2.5 μl RLT Plus lysis buffer (Qiagen, 1053393) and 1 U μl^−1^ SUPERase In RNase Inhibitor (Invitrogen, AM2694). Samples were immediately frozen on dry ice and kept at −80 **°**C until further processing.

For barcoding experiments, intact CTC clusters were picked as described above and deposited into individual PCR tubes containing 1 μl of oligo-dT primer, 1 μl of dNTP mix and 2.3 μl of cell lysis buffer (0.2% (vol/vol) Triton X-100 (Sigma-Aldrich, X-100) and 2 U μl^−1^ SUPERase In RNase Inhibitor). Samples were immediately frozen on dry ice and transferred to −80 °C until further processing. All pre-PCR steps were carried out in a PCR cabinet with laminar air flow to reduce environmental contamination.

### Primary tumor processing

Barcoded primary tumors were surgically resected from mice after terminal blood sampling, transferred to 50 ml screw-cap tubes (Sarstedt, 62.547.254) containing precooled CO_2_-Independent Medium (Gibco, 18045-088) and stored on ice until further processing. Subsequently, tumor tissue was transferred to Lysing Matrix S tubes (MP Biomedicals, 116925500) and homogenized on a Precellys 24 tissue homogenizer (Bertin Technologies) for 2 × 20 s at 5,500 rpm. Homogenized tumor tissue was transferred to 50 ml screw-cap tubes and lysed in 18 ml of tissue lysis buffer (40 mM TRIS pH 8, 1% SDS and 50 mM EDTA) supplemented with 100 μl Proteinase K (Qiagen, 19133) with constant shaking at 55 °C overnight. The next day, 100 μl of 100 mg ml^−1^ RNAse A (Qiagen, 19101) was added; tubes were thoroughly mixed through inversion and incubated with constant shaking for 30 min at 37 °C. After that, tubes were immediately chilled on ice before the addition of 9 ml precooled 7.5 M ammonium acetate solution (Sigma-Aldrich, A2706), followed by thorough mixing through inversion of tubes and rigorous vortexing for 1 min at full speed to reduce the molecular weight of DNA. Subsequently, the tubes were centrifuged at 4,400*g* for 10 min at 4 °C to precipitate salts and proteins. DNA was recovered from the supernatant by decanting on top of 20 ml of 100% isopropanol in a fresh 50 ml tube. Tubes were mixed by inversion 50 times and centrifuged at 4,400*g* for 15 min at 4 °C to pellet DNA. Supernatants were discarded and DNA pellets were purified twice with precooled 70% ethanol. Ethanol was removed and DNA pellets were dissolved in TE buffer (Invitrogen, 12090015) over constant agitation. Dissolved DNA samples were sheared in 1 ml AFA Fiber milliTUBEs (Covaris, 520135) on a LE220-plus instrument (Covaris) for 60 s with 200 cycles per burst, a duty factor of 10% and a peak incident power of 450 to reduce the molecular weight of DNA and increase PCR efficiency.

### Exome sequencing

Exome sequencing of CTC samples was performed based on the previously published G&T-seq protocol^29^. Genomes and transcriptomes of lysed cells were separated, and genomes were amplified using the GenomiPhi V3 Ready-To-Go DNA Amplification Kit (Cytiva, 25-6601-97). Libraries were prepared using the Nextera XT DNA Library Preparation Kit (Illumina, FC-131-1096); exomes were enriched using the SureSelect XT Human All Exon v6 + Cosmic Kit (Agilent Technologies, 5190-9308) and sequenced on a HiSeq 2500 instrument (Illumina) in 100 bp paired-end mode.

### Exome sequencing analysis

Paired-end reads were aligned to the GRCh38 human reference using BWA-mem algorithm (v0.7.15)^30^ and sorted using SAMtools (v.1.7)^31^. Xenograft samples were additionally aligned to the GRCm38 mouse reference genome and assigned to either human or mouse using Disambiguate (v1.0.0)^32^. Reads identified as mouse were removed from subsequent analysis. Deduplication of reads was performed on a per-sample basis using Picard MarkDuplicates (v.2.9.2), and local realignment was performed using the Genome Analysis Toolkit IndelRealigner (v.3.7.0)^33^ at the sample and donor level to improve alignment accuracy around indels. Quality control as well as coverage and exome enrichment statistics were generated using FastQC (v.0.11.8), CollectHsMetrics from Picard suite (v.2.9.0) and QualiMap (v.2.2.1)^34^ and visualized using MultiQC (v.0.8)^35^. Mpileup files were generated with SAMtools (parameters: -q 40 -Q 30) at donor level, and variants were called using SCIΦ on all samples from the same donor simultaneously.

### Genetic variant annotation

The variant annotation and effect prediction tool SnpEff (v.5.2a)^36^ was used to classify observed genetic variants by putative impact on protein functionality, using default parameters and variant calling format files as input. The Cancer Genome Interpreter web tool was used to analyse genetic variants by their predicted oncogenic capacity^37^.

### Barcode sequencing

Amplified cDNA was obtained for individual CTC cluster samples following the previously published SmartSeq2 protocol^38^. Barcode loci were amplified from purified cDNA (CTC cluster samples) or sheared gDNA (primary tumor samples) using the KAPA HiFi HotStart ReadyMix (Kapa Biosystems, KK2602) supplemented with 5% (vol/vol) DMSO and a set of equimolar pools of staggered primers flanking the barcode locus (final concentration of pools = 300 nM), following cycling conditions according to the manufacturer's recommendations with a primer annealing temperature of 63.5 °C. Barcode amplicon samples were then submitted to a second PCR step to introduce unique dual indexes, sequencing primer-binding sites and Illumina adapter sequences P5 and P7. All primers used are listed in Supplementary Table 3. All PCR steps were performed in a T100 Thermal Cycler (Bio-Rad). Final amplicons were purified using AMPure XP Beads (Beckman Coulter, A63881) and sequenced on an Illumina NovaSeq instrument in 150-base-pair paired-end mode to generate files in FASTQ format.

### Barcode analysis

Reads in FASTQ files were aligned to barcode reference sequences using bowtie2 (v2.5.1; parameters: --local --score-min L,130,0), considering only reads aligning in full length without mismatch. Resulting SAM files were sorted using Samtools sort (v1.16.1), and the number of read segments mapped to each barcode reference sequence was counted using Samtools idxstats (v1.16.1). The resulting barcode count files were processed in R (v4.2.3, R Foundation for Statistical Computing) for secondary analyses. Taking into consideration an expected single barcode integration event per cell, samples were removed from downstream analyses when the smallest number of distinct barcodes accumulating 90% of total aligned reads was higher than the expected number of cells in the sample, indicating profound background noise contribution as seen in negative control samples. CTC cluster samples were classified as monoclonal or oligoclonal based on the detected barcode distribution, taking into consideration the read count of the most abundant barcode relative to the second most abundant barcode and the number of cells in the corresponding CTC cluster sample. A CTC cluster was determined to be monoclonal whenever the read proportion of the most dominant barcode exceeded the read proportion of the second most abundant barcode multiplied by the number of cells in the cluster. Otherwise, the CTC cluster was determined to be oligoclonal.

### Statistics and reproducibility

Statistical testing and visualizations were conducted in R (v4.2.3, R Foundation for Statistical Computing). Graphical illustrations in Figs. 1a–c and 2a and Extended Data Figs. 2a and 3b were generated using BioRender.com and Adobe Illustrator. No statistical method was used to predetermine sample size. For mouse experiments, sample sizes were determined in accordance with the 3R principles and consistent with those reported in previous publications^13,24^. No data were excluded from the analyses. All mice were randomized before experiments and blindly selected before tumor cell injection. Two independent animal experiments were performed, confirming the reproducibility of our findings.

### Reporting summary

Further information on research design is available in the Nature Portfolio Reporting Summary linked to this article.

## Online content

Any methods, additional references, Nature Portfolio reporting summaries, source data, extended data, supplementary information, acknowledgements, peer review information; details of author contributions and competing interests; and statements of data and code availability are available at 10.1038/s41588-025-02205-2.

## Supplementary information

Supplementary Information Supplementary Notes 1–4 and Figs. 1–4.

Reporting Summary

Supplementary Tables 1–4 Supplementary Table 1: Clinical characteristics of interrogated patients with cancer. The table showing the sex and age (years) at primary cancer diagnosis, tumor stage and grade, histologic subtype (for patients with breast cancer—percentage of estrogen receptor-positive cells, percentage of progesterone receptor-positive cells, percentage of Ki67-positive cells, HER2 amplification status (positive, negative)), single CTC, CTC cluster and CTC-white blood cell cluster counts per 7.5 ml of blood, sites of metastasis, and number and type of previous systemic treatment lines. ER, estrogen receptor; PR, progesterone receptor; nd, not done; na, not applicable. Supplementary Table 2: Statistics on interrogated CTC clusters from barcoded xenograft samples. The table showing for each xenograft sample the number of engrafted barcoded cancer cells, the complexity level of resulting primary tumors, the total number of included CTC clusters, as well as the number of examined CTC clusters with two and three or more cells. Supplementary Table 3: List of primers used for barcode amplification. The table showing oligonucleotide sequences. Supplementary Table 4: European Nucleotide Archive accession codes for sequencing datasets initially included in ref. ^13^*.* The table showing European Nucleotide Archive accession codes, as well as aliases and titles for samples initially included in ref. ^13^.

## Source data

Source Data Fig. 1 Statistical source data.

Source Data Fig. 2 Statistical source data.

## Data Availability

The sequencing datasets that support the findings of this study have been deposited in the European Nucleotide Archive (ENA, EMBL-EBI; PRJEB77733). The sequencing datasets for samples initially included in ref. ^13^ are deposited under ENA accession PRJEB24623 (Supplementary Table 4). The genome references used in this study were obtained from Gencode (https://www.gencodegenes.org/human/release_32.html for GRCh38 and https://www.gencodegenes.org/mouse/release_M24.html for GRCm38). Source data are provided with this paper.

## Code availability

Original code to reproduce the phylogenetic analysis, as well as the analysis of barcoded xenograft samples, has been deposited to GitHub (https://github.com/cbg-ethz/CTC-SCITE) under the GPL-3.0 license and archived at Zenodo (10.5281/zenodo.12774098)^39^.
