# Supplementary material for: Phylogenetic inference reveals clonal heterogeneity in circulating tumor cell clusters
Source: Nat Genet. 2025 Jun 2;57(6):1357–61. doi: 10.1038/s41588-025-02205-2 (PMC12165839; doi:10.1038/s41588-025-02205-2)
Supplement: Supplementary file 1 — Supplementary Notes 1–4 and Figs. 1–4. [file 41588_2025_2205_MOESM1_ESM.pdf]

nature genetics

Supplementary information https://doi.org/10.1038/s41588-025-02205-2

Phylogenetic inference reveals clonal heterogeneity in circulating tumor cell clusters

In the format provided by the authors and unedited

Supplementary Note 1:
Inference of CTC and CTC cluster genealogies to study oligoclonal metastatic seeding

Circulating tumor cells (CTCs) are shed from solid tumor lesions into the blood stream and carry the genetic profile of their clone of origin. Therefore, we can gain insights into the clonal composition of the CTC-disseminating areas of the primary tumor and metastases by sequencing CTCs. CTCs are often shed into the bloodstream as clusters of two or more physically associated cells and frequently admix with tumor-associated cells like white blood cells (WBCs). When these clusters cannot be physically broken into single cells without harming cell integrity, the joint DNA material of all cells in the cluster is sequenced in a single sample, resulting in an aggregate set of variants of the CTC cluster. We developed a pioneering computational method to deconvolve the aggregate read count profiles of CTC clusters. Using these profiles along with single CTCs, we jointly infer the genealogy of all sampled cells, as well as their individual genotypes. The clusters are thereby split computationally into their constituent cells in the tree, and the resulting genotypes of the single cells allow us to assess clonality of the CTC clusters. The model builds on our existing tree inference algorithms for single cell data SCITE and SCIΦ. However, here we model the aggregated nature of cells in CTC clusters. To our knowledge, this is the first computational approach to perform explicit genetic deconvolution of aggregated single cells.

Model overview

We consider the CTC genealogy, a leaf-labelled binary tree T in which the leaves correspond to single CTCs. Whenever a progenitor cell produces mutated offspring, this mutation event is placed on the edge connecting these cells (Supplementary Fig. 1). We constrain the space of genealogies to an infinite sites model, which assumes that each mutation occurs at most once throughout the whole process of somatic cell evolution and is passed on to all descendant cells. This assumption effectively restricts our model to heterozygous mutations, as a homozygous mutations would require two genomic alteration events on the same genomic site. A proposed genealogy yields an aggregate genotype for each CTC cluster and single cell, which can be compared to read data. This allows us to determine how well a genealogy, including mutational placements and splitting of CTC clusters, fit to total and variant read counts of all CTCs and CTC clusters.

We use a Markov Chain Monte Carlo (MCMC) approach to perform Bayesian inference of the tree topology. More precisely, our algorithm takes total and variant read counts of CTCs and CTC clusters as input and outputs a set of tree topologies, where the number of the trees in the output set is proportional to their posterior probability. Each edge-labelled tree deconvolves the aggregate profiles of the CTC clusters. The tree may place cells from the same CTC cluster onto different branches, indicating that the genotypes of the component cells differ.

For each output tree and CTC cluster, we compute a *splitting score*, reflecting the probability that the cells of the cluster originate from genetically distinct lineages, and average this probability over all output trees. Thus, the splitting score allows us to make inference about the clonality of a CTC cluster while accounting for the uncertainty in tree reconstruction. We also derive a consensus of each cell's individual genotype to identify mutations that are unique for specific cells of the CTC cluster and annotate them according to their functional impact on protein activity.

Methods

Input Data

The model takes as input two matrices K and R with n' rows labelled by mutation sites and m columns labelled by CTCs and CTC clusters. The observed mutated and total read counts $k_{i,\ell}$ and $r_{i,\ell}$ are encoded in the $n' \times m$ matrices K and R and summarized as $D = (K, R)$. Additionally, for each CTC cluster, the number of tumor cells and WBCs is specified.

Multiple displacement amplification model

Single-cell DNA sequencing requires initial amplification of the available DNA material, such as Multiple Displacement Amplification (MDA). In this approach, a newly synthesized fragment becomes immediately available as a template for further amplification along with its own template. This results in a "rich gets richer" phenomenon, where fragments that happened to be among the first to be amplified end up over-represented in the final read count distribution. In particular, heterozygous mutations can produce a read count pattern where the fraction of variant reads is far from the expected 50%.

We account for this over-dispersion by employing a beta-binomial distribution akin to the Pólya urn model that describes an experiment where a ball that is drawn in one round is replaced into the urn along with an additional copy for the next round. Let r be the coverage at a sequence position, α the number of variant alleles prior to amplification, and β the number of wild type alleles prior to amplification. Then the probability of obtaining k reads through MDA is

$$P_{BBin}(k \mid r, \alpha, \beta) = \binom{r}{k} \frac{B(k+\alpha, r-k+\beta)}{B(\alpha, \beta)}$$

where B is the beta function. The total number of variant and wild type alleles depends only on the genotype in each of the cells (Supplementary Fig. 1).

Allelic dropout model

In addition to over-dispersion, MDA is also prone to allelic dropout, the process in which some alleles are randomly not amplified at all. To account for this issue, we introduce a dropout rate δ, which defines, for each allele, the probability to not partake in the amplification process. An example is given in Supplementary Fig. 2.

Since we do not distinguish between alleles of the same type, the probability that p mutated alleles drop out and q normal alleles drop out is the product of two binomial distributions,

$$P_{Bin}(p \mid \alpha, \delta) P_{Bin}(q \mid \beta, \delta) = \binom{\alpha}{p} \binom{\beta}{q} \delta^{p+q} (1-\delta)^{\alpha+\beta-(p+q)}$$

We do not know the number of dropped out alleles, so we marginalize over its distribution, i.e., we condition the read count distribution on the dropout and sum over all possible values of $p = 0, \dots, \alpha$ and $q = 0, \dots, \beta$, except the case $p = \alpha$ and $q = \beta$, as no reads would then be produced. In this case, we set the probability of observing reads to 0.

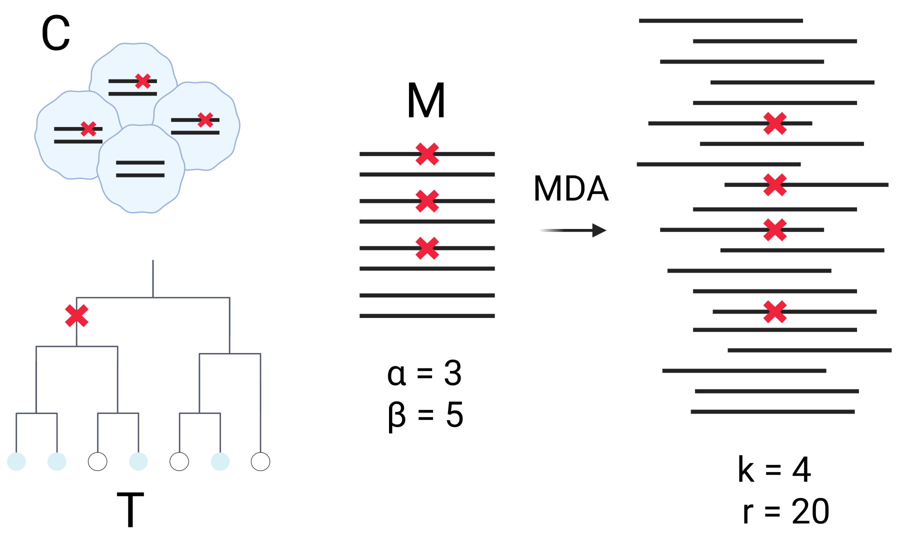

Supplementary Fig. 1. Example of oligoclonal four-cell cluster. Three cells exhibit a (heterozygous) mutation M (red cross) and the fourth does not, which leads to a 3:5 ratio of variant and wild type alleles, i.e., a variant frequency of $\mu = 37.5\%$. Accordingly, three out of the four cells are located in the subtree below a M. After MDA, the read count fractions have shifted away from 37.5% to $4 : 16 = 25\%$ due to random amplification bias. The figure was created using BioRender.com.

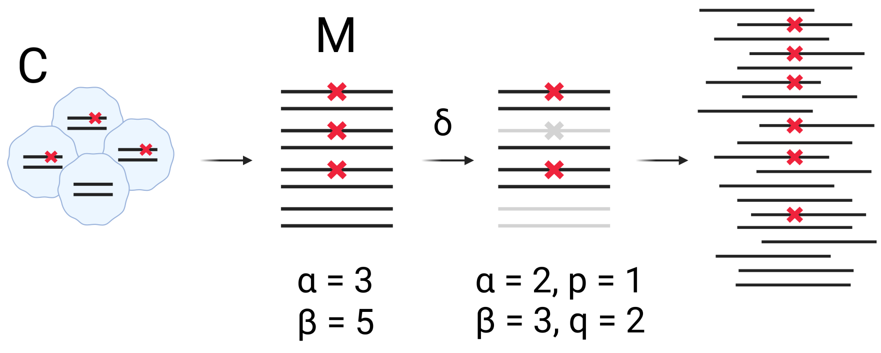

Supplementary Fig. 2. Dropouts in an oligoclonal four-cell cluster. Each allele is modelled to have a probability δ to drop out, i.e., to not partake in the amplification process. In this example, instead of starting the MDA process with a 3:5 ratio of variant and wild type alleles, $p = 1$ mutated allele and $q = 2$ wild type alleles drop out (shown in grey), shifting the initial parameters for MDA to $\alpha = 2$ and $\beta = 3$. The figure was created using BioRender.com.

Sequencing error model

A third type of disturbances of read count fractions are sequencing errors. Sequencing takes place after amplification, and sequencing errors are much rarer than the other two types of errors, so we only consider them in cases where all alleles of one type have been lost such that the respective allele can only be introduced by sequencing errors.

We assume that each wild type read is sequenced as mutated and vice versa independently at a global error rate ε. The probability to produce k mutated reads from r wild type reads through sequencing errors follows a binomial distribution $P_{Bin}(k \mid r, \varepsilon)$. Likewise, $P_{Bin}(r-k \mid r, \varepsilon)$ denotes the probability to produce $r-k$ wild type reads from r mutated reads solely through sequencing errors. Then, under the assumption of independence of dropout events and sequencing errors, the joint probability that all wild type alleles drop out and not all mutated alleles drop out and that $r-k$ wild type reads are produced from r reads is

$$P_{Bin}(\beta \mid \beta, \delta)(1 - P_{Bin}(\alpha \mid \alpha, \delta))P_{Bin}(r-k \mid r, \varepsilon) = \delta^{\beta}(1-\delta^{\alpha})\binom{r-k}{k}\varepsilon^{r-k}(1-\varepsilon)^{k}$$

Symmetrically, the joint probability that all mutated alleles drop out and not all wild type alleles drop out and that k mutated reads are produced from r reads is obtained by swapping α and β in above formula and replacing $r-k$ by k.

Read count model

Combining all three model parts, we obtain the full read count model for observing k mutated reads:

$$\begin{aligned} P_R(k \mid r, \alpha, \beta, \delta, \varepsilon) = &\left[\sum_{p=0}^{\alpha-1}\sum_{q=0}^{\beta-1} P_{Bin}(p \mid \alpha, \delta)P_{Bin}(q \mid \beta, \delta)P_{BBin}(k \mid r, \alpha-p, \beta-p)\right] \\ &+ P_{Bin}(\beta \mid \beta, \delta)(1 - P_{Bin}(\alpha \mid \alpha, \delta))P_{Bin}(r-k \mid r, \varepsilon) \\ &+ P_{Bin}(\alpha \mid \alpha, \delta)(1 - P_{Bin}(\beta \mid \beta, \delta))P_{Bin}(k \mid r, \varepsilon) \end{aligned}$$

Probabilistic tree model

Let C_ℓ denote the set of cells in sample l. In total, we have $n = \sum_{i=1}^{n'} |C_\ell|$ cells. Let m denote the number of mutated sites over all cells. We describe the cell genealogy as a binary leaf-labelled tree T with n leaves. Each leaf is associated with exactly one sample, while a sample can be associated with multiple leafs (indicating individual cells of CTC clusters). T is augmented by a vector $\sigma = (\sigma_1, \ldots, \sigma_m)$ whose i-th entry indicates where mutation M_i is placed in the tree. We derive the expected variant and wild type allele counts $\alpha_{i,\ell}$ and $\beta_{i,\ell}$ for a given sample C_ℓ and mutation M_i by counting how many cells of C_ℓ descend from M_i in the tree.

We compute the likelihood to observe the read count data given a tree T augmented by the mutation placement σ and model parameters $\theta = (\delta, \varepsilon)$ as

$$P(D \mid T, \sigma, \theta) = \prod_{i=1}^{m}\prod_{\ell=1}^{n'} P_R(k_{i\ell} \mid r_{i\ell}, (\alpha_{i\ell})_{T,\sigma}, (\beta_{i\ell})_{T,\sigma}, \theta) \tag{1}$$

For the posterior distribution, we apply Bayes' theorem, and we marginalize out the attachment points of the mutations, akin to [1]:

$$
\begin{aligned}
P(T,\theta \mid D) &= \sum_{\sigma} P(T,\sigma,\theta \mid D) \\
&\propto \sum_{\sigma} P(D \mid T,\sigma,\theta) P(T,\sigma,\theta) \\
&\propto \sum_{\sigma} P(D \mid T,\sigma,\theta) P(\sigma \mid T,\theta) P(T,\theta) \\
&= \sum_{\sigma} \prod_{i=1}^{m} \prod_{\ell=1}^{n} P_R(k_{i\ell} \mid r_{i\ell}, (\alpha_{i\ell})_{T,\sigma}, (\beta_{i\ell})_{T,\sigma}, \theta) P(\sigma_i \mid T,\theta) P(T,\theta) \\
&= \prod_{i=1}^{m} \sum_{\sigma_i} \prod_{\ell=1}^{n} P_R(k_{i\ell} \mid r_{i\ell}, (\alpha_{i\ell})_{T,\sigma}, (\beta_{i\ell})_{T,\sigma}, \theta) P(\sigma_i \mid T,\theta) P(T,\theta)
\end{aligned}
$$

In the last line, the terms were rearranged, such that each mutation can be placed and evaluated independently, which reduces the size of the search space from $(2n-1)^m(2n-3)!!$ to $(2n-3)!!$ [2]. This is possible as long as the prior factorizes as $P(\sigma \mid T,\theta) = \prod_{i=1}^{m} P(\sigma_i \mid T,\theta)$.

Prior distributions

Some of the CTC clusters contain WBCs. We assume that WBCs have lower mutational burden compared to cancer cells; hence they should be attached close to the root of the tree. To account for this, we penalize the cell attachment of WBCs proportionally to the inferred genetic distance of the WBC from the root. In probabilistic terms, we define the prior probability $P(\sigma_i \mid T,\theta)$ to be proportional to $\exp(-h_{\sigma_i})$, where h_{σ_i} is the number of WBCs descending from the position of mutation attachment σ_i. For the joint prior distribution of tree and model parameters $P(T,\ \theta)$, we choose a uniform prior on the parameter space.

Inference

We explore the parameter space of trees T and dropout and error rates $\theta = (\delta,\ \varepsilon)$ with an MCMC approach to sample from the posterior distribution $P(T,\theta \mid D)$. We use the Metropolis-Hastings algorithm to generate a sequence of trees by iteratively changing one randomly chosen parameter at a time, according to a proposal distribution.

We specify the tree moves akin to [1] as follows: The rearrangement by subtree pruning and regrafting selects a node uniformly at random, removes the subtree defined by the descending nodes from the tree, and attaches this subtree to an edge uniformly chosen from the remaining tree [2]. Node swapping selects two nodes of the tree uniformly at random and swaps them together with their subtrees. The error parameters are explored through a Gaussian random walk with standard deviation of 0.1 centered around the current value.

We compute the acceptance probability at which the new parameter configuration becomes the next state of the chain (otherwise the old configuration is kept). The resulting Markov Chain is ergodic, such that its stationary distribution is guaranteed to be the posterior distribution of the model. Asymptotically, the Markov Chain generates a set of trees sampled from the posterior distribution.

Assessing the clonality of a CTC cluster

We seek to determine if a CTC cluster contains two cells from independently evolving lineages. In terms of the underlying tumor phylogenetic tree, we ask whether the CTC cluster splits up into cells that are placed on different branches of the trees, i.e., whether there are mutations on both branches separating the cells and connecting them to their most recent common ancestor.

The main idea to answer this question is the following: Given a CTC cluster, we define a *splitting score* which measures how much evidence a tree provides for splitting the cluster. We sample trees from the posterior distribution as described above, derive the score for each tree, and then average the score over the set of trees. We use this score as a test statistic, and assess its null distribution from simulated monoclonal CTC clusters. Average splitting scores significantly larger than what we expect from monoclonal CTC clusters are thus an indication for oligoclonal CTC clusters.

Specifically, given a tree T, we account for the uncertainty of the mutation placement by considering each mutation's probability of mapping to either of two branches. The cluster splits with low probability if for one of the branches it is unlikely that any mutation maps to it. On the other hand, it splits with high probability if for each of the branches there exists a mutation likely mapping to it (Extended Data Fig. 2b). The following splitting score encodes this:

$$S_T(c_1, c_2) \coloneqq \min_{j=1,2} \max_{1\leq i\leq m} P(M_i \text{ maps to } b_j)$$

where c_1 and c_2 are the cells associated to the same CTC cluster, and b_j is the path from c_j to the most recent common ancestor of both cells, for $j = 1, 2$.

To additionally account for the uncertainty in the tree inference, we compute the splitting score for each sampled parameter and tree and form the average. Since the parameters are sampled from the posterior distribution, the splitting score is (approximately) weighted by the posterior probability of the parameters. The average splitting scores is

$$S(c_1, c_2) = \int_{T,\theta} S_T(c_1, c_2) dP(T, \theta \mid D)$$

A high value relative to the null distribution suggests that the data is unlikely to be generated by a pair of cells of monoclonal origin (Extended Data Fig. 2a).

Simulation of monoclonal CTC clusters

We simulated monoclonal CTC clusters to approximate the null distribution of the average splitting score in the absence of oligoclonality.

As a first step, we identified a set of genotypes that is consistent with the phylogeny by using the tree's output to call the genotypes of single cells in the dataset as done in [1], and averaged the calls over all sampled trees. These genotypes served as templates for our simulations. This step was necessary to ensure that the genotype of a simulated monoclonal CTC cluster matches the underlying true genealogy, as genotypes that cannot be explained by the true genealogy are likely to bias the tree reconstruction towards incorrect genealogies.

In our simulation, each allele may be removed at equal rate. The model above does not describe the distribution of total read counts. Therefore, we assessed the empirical distribution of total read counts given by the matrix R by fitting a zero-inflated negative-binomial distribution. The total reads for a simulated CTC cluster were drawn from this distribution. Mutated read counts were produced by a beta-binomial distribution. Non-mutated reads were randomly be converted to mutated reads and vice versa. Dropouts occured at a rate of 0.35 and errors at a rate of 0.0015, chosen according to the MAP estimate of the posterior sampling. For each dataset, we simulated four 2-cell CTC clusters, three 3-cell CTC clusters, two 4-cell and two 5-cell CTC clusters.

We assess the posterior distribution of trees including simulated CTC clusters and derive the distributions of $S_T(c_1, c_2)$. For each cluster size, we aggregated these distributions to obtain cluster size-specific null distributions. A CTC cluster is considered oligoclonal, if the value $S(c_1, c_2)$ exceeds the 95%-quantile of the empirical null distribution for at least one pair of cells (c_1, c_2) associated to the cluster.

Identifying individual genotypes in a CTC cluster

Exchanging two cells from the same cluster in the tree gives rise to the exact same inferred aggregate genotype of the CTC clusters. Since the likelihood (Eq. 1) depends only on this aggregate genotype and not the genotypes of the individual cells, the tree model cannot distinguish between two tumor cells of the same CTC cluster. We leveraged this fact to define a co-occurrence distance metric on the set of mutations. Given a CTC cluster, a private branch is defined as the path from a leaf belonging to the cluster to the node just below the most recent common ancestor of all cells in the cluster. Given a tree, two mutations M_1 and M_2 either do or do not co-occur on any of the private branches. We therefore define the co-occurrence distance

$$d(M_1, M_2) \coloneqq 1 - \frac{\#\text{co-ocurrences of } M_1 \text{ and } M_2 \text{ in a private branch}}{\#\text{sampled trees}}$$

Each CTC cluster found oligoclonal gives rise to a matrix of distances among all mutations. After filtering mutations distant to all other mutations, we clustered mutations hierarchically by average linkage and cut the resulting dendrogram to obtain at most as many clusters as there were cells in the CTC cluster. Since the clustering signal was typically very strong, we determined the number of clusters by visual inspection (Supplementary Fig. 3).

The clustered mutations indicate the genetic differences separating cells of the same CTC clusters. To interpret these differences further, mutations in the clusters were annotated using SnpEff [3], and the number of variants with high annotation impact and with moderate annotation impact were counted.

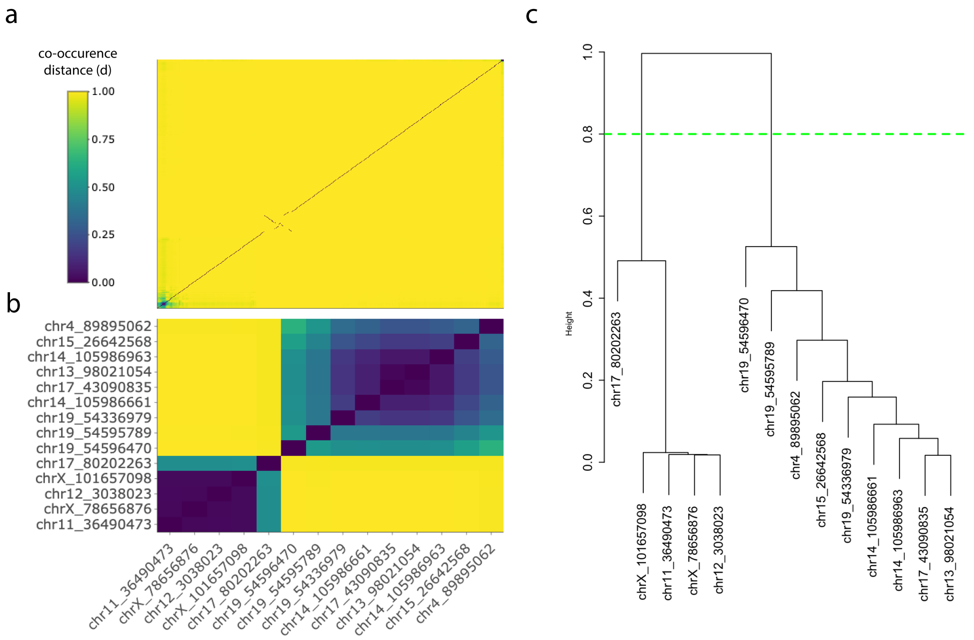

Supplementary Fig. 3. Representative clustering of genes for a two-cell CTC cluster. a, Distance matrix of all mutations for the distance metric d, indicating how often mutations co-occur on the same branch of the tree throughout tree sampling, represented by a heatmap. **b,** Distance matrix after filtering variants that are distant to all others. The CTC cluster does likely not carry these variants. **c,** Dendrogram on the filtered mutations exhibits two distinct clusters of variants.

CTC cluster annotation

We used the following annotation of CTC clusters: We considered CTC clusters to show evidence for branching evolution, if the splitting score exceeds the defined threshold. Moreover, we infered mutations to distinguish the different genotypes as described above and categorized CTC clusters with evidence for branching evolution according to the functional impact of these separating mutations.

Supplementary Results: Simulation-based performance assessment of the tree algorithm

To characterize the overall ability of the tree-based algorithm to determine the oligoclonality status of CTC clusters and its deconvolution performance, we simulated monoclonal CTC clusters as described above, and oligoclonal CTC clusters by aggregating the read counts from randomly sampled genetically distinct single tumor cells (Supplementary Fig. 4). For each of these simulated clusters as well as the non-aggregated genetically distinct single cells, we computed the mean splitting scores averaged over all samples from the posterior distribution (sampling size = 1000, Supplementary Fig. 4). Our comparison shows that simulated monoclonal CTC clusters consistently exhibit a low mean splitting score with a mean of 0.074, a median of $8.97 \cdot 10^{-4}$ and a 90%-percentile of 28.6%, while for simulated oligoclonal clusters the mean splitting scores were elevated with a mean of 0.64, a median of 0.66 and a 10%-percentile of 0.37. This shows that the splitting score is strongly indicative of the clonality status of the CTC clusters. With a mean of 0.73 and a median of 0.94, the splitting scores of single tumor cells are higher than for simulated oligoclonal clusters. An increase in performance with respect to CTC clusters is to be expected, since the tree inference algorithm can additionally exploit information

of co-occurrence of mutations obtained from single-cell exomes. The splitting score of the simulated monoclonal clusters has a standard error of 0.17 and is lower compared to 0.22 and 0.37 for simulated oligoclonal CTC clusters resp. single cells. This can be explained by varying degrees of genetic dissimilarity of the constituent cells of simulated oligoclonal CTC clusters, which may provide a differing amounts of evidence for oligoclonality. Our analyses proved that the tree-sampling based deconvolution in combination with the splitting score is a suitable instrument to distinguish monoclonal CTC clusters from oligoclonal CTC clusters.

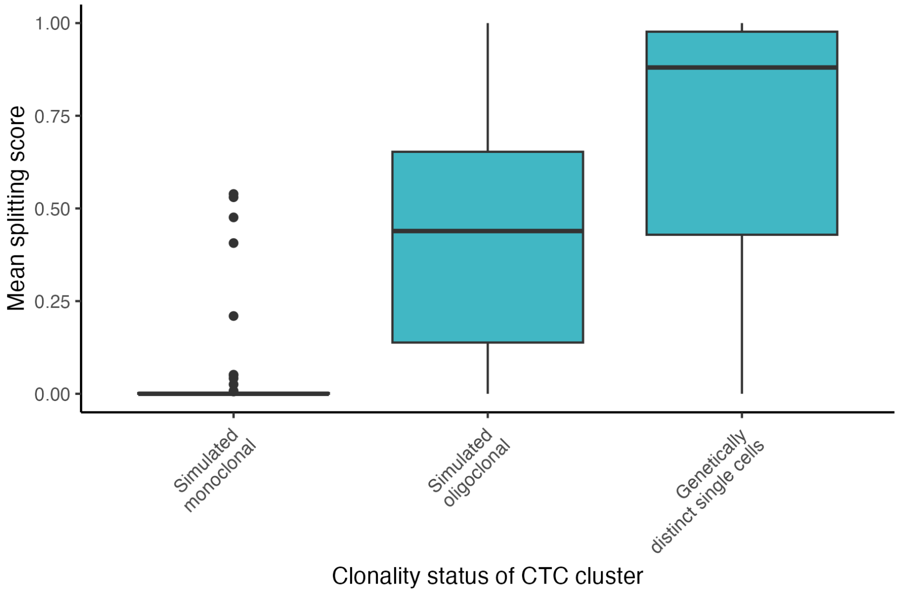

Supplementary Fig. 4. Simulation study to asses the performance of the phylogenetic tree-based assessment of oligoclonality. Boxplots show the empirical distributions of mean splitting scores computed for 50 simulated monoclonal CTC clusters, simulated oligoclonal CTC clusters, and genetically distinct single tumor cells, respectively. A higher mean splitting score indicates stronger evidence for oligoclonality. The centers of the boxplots are defined as the medians of the estimates, upper and lower hinges show the first and third quartiles, respectively, and whiskers reach out to the furthest points whose distance from the hinges is smaller than 1.5 times the inter-quartile range. All outliers are plotted as points.

Supplementary Note 2: Simulation of the proportion of unique barcodes within cell pools for *in vivo* engraftment to model clonal expansion

We sought to model clonal expansion in vivo through orthotopic engraftment of uniquely barcoded cancer cells, ensuring that the duplicate barcode fraction is low in the engrafted cell population. To estimate the proportion of unique barcodes within cell pools of a given complexity, we simulated our experimental design of clonally labeling LM2 breast cancer cells with molecular barcodes as follows.

We generated barcode read count data based on next-generation sequencing (NGS) quality control (QC) data of the Clonetracker XP barcode library (provided by manufacturer) in R to obtain a pool of barcode IDs, where each barcode is represented according to the NGS read distribution. We randomly sampled a number of observations from the generated pool of barcode IDs, reflecting increasing numbers of initially transduced cells (between 10^4 and $5 \cdot 10^5$ cells). We then replicated the sampled barcode IDs eight times, simulating three rounds of cell replication within the 72h period between transduction and cell engraftment (considering an approximate cell doubling time of 24 h for the LM2 cell line). From the replicated pool of barcode IDs, we then randomly sampled a number of barcodes corresponding to the number of cells for in vivo engraftment (between 10^2 and $5 \cdot 10^4$ cells) and evaluated the proportion of unique barcode IDs in the final sample.

For each combination of initially transduced cells and final cell pool for engraftment, we report the mean proportion of unique barcodes and the standard deviation after resampling ten thousand times (Extended Data Fig. 3c).

Supplementary Note 3: Measuring distortion in the representation of clones among CTC clusters

Next, we measured the presence of clones in CTC clusters from xenograft models with clonally barcoded primary tumors and compared the relative abundance of clones among CTC clusters with their measured frequency in the primary tumor. To gain a clearer insight into the process of formation and selection of CTC clusters intravasating the blood stream, we took as a basis a model according to which the probability of a CTC originating from a clone is proportional to the prevalence of that clone in the primary tumor. Since a CTC cluster can contain cells from at most as many different clones as it consists of cells, it could not be assumed that the presence of different clones in the same CTC cluster are independent. Let r_j be the size of CTC cluster j, t be the number of clones in the primary tumor and assume that we know the vector $f := (f_1, \ldots, f_t)$ of the (strictly positive) proportions of all clones. We formulated the null hypothesis that the number of times that each of the clones appear in cluster j is multinomially distributed with r_j trials and the probability vector $(f_1, \ldots, f_t)$. We derived and applied a statistical test to detect when the clonal composition of CTC clusters could not be explained by random sampling.

For this, we additionally assumed that the proportions of clones in the primary tumor correspond exactly to the proportions of measured barcode counts. However, the RNA-sequencing based barcode counts were too noisy as to provide a reliable estimate of the exact number of cells in a CTC cluster belonging to a specific clone. To avoid making additional assumptions, we were agnostic towards to exact clonal composition of the CTC clusters. Instead, we measured the presence ($d_{ij} = 1$) or absence ($d_{ij} = 0$) of clone i in cluster j as a binary variable. Treating the multinomially distributed clonal composition as a latent variable, the expected indication of d_{ij} is given by

$$E_{ij} = 1 - (1 - f_i)^{r_j}$$

We defined a test statistic, akin to the classical G-test statistics [4]:

$$G_j = 2 \sum_{\substack{i=1 \\ d_{ij} \neq 0}}^{t} d_{ij} \log \left(\frac{d_{ij}}{E_{ij}} \right)$$

For each CTC cluster size and mouse model, the distribution of G_j was simulated with a sampling size of 10000 and defined the p-value of a single CTC cluster as the probability of observing a G-score at least as high as that of the CTC-cluster. To obtain a final p-value, we assumed independence of G_j between different CTC clusters and combined their p-values using Fisher's method.

Supplementary Note 4:
Quantitative assessment of monoclonality in CTC clusters

Furthermore, we used the xenograft models to measure the prevalence of oligoclonal CTC clusters and associate it with cluster size and primary tumor complexity. From this model, we derived and applied a statistical test to detect if more CTC clusters were found monoclonal than expected under the multinomial model from Supplementary Note 3.

Let t be the number of clones in the primary tumor and assume we know the vector $f = (f_1, \ldots, f_t)$ of proportions of all clones. Then the probability of a CTC cluster j of size r_j to be monoclonal is

$$P(f) = \sum_{i=1}^{t} f_i^{r_j}$$

We modelled the random selection of a CTC cluster from the liquid biopsy akin to a random draw from an urn filled with balls of two different colors (representing monoclonal vs oligoclonal clusters) with replacement. This amounts to saying that the number of monoclonal CTC clusters is binomially distributed where the number of trials is the number of CTC clusters of a certain size and the success probability is given by $P(f)$. This binomial distribution defines our null distribution. The test measures how likely it is to observe a given number of monoclonal CTC clusters or more.

We estimated the vector f from the barcode counts. We assumed that the likelihood of observing a barcode count configuration given some clonal proportions is multinomially distributed. Assuming a uniform prior distribution of primary tumor clone proportions, and given a vector of absolute barcode counts $(s_1, \ldots, s_t)$, the posterior probability distribution is Dirichlet distributed with parameter vector $(1+s_1, \ldots, 1+s_t)$. To compute the p-value of the statistical test, we marginalized out the latent vector f. The p-value was then computed as

$$\int_f \binom{n}{k} \left(\sum_{i=1}^{t} f_i^{r_j}\right)^k \left(1 - \sum_{i=1}^{t} f_i^{r_j}\right)^{n-k} P_{Dir}(f, (1+s_i)_i)df$$

where the integral was taken over the parameter space

$$\left\{ (f_i)_{1 \le i \le t} \;\middle|\; \sum_{i=1}^{t} f_i = 1 \text{ and } f_i \in \mathbb{R}_{\ge 0} \right\}$$

We approximated the integral by Monte-Carlo integration, through sampling of the parameter f.

Supplementary References

[1] Singer, J., Kuipers, J., Jahn, K. *et al.* Single-cell mutation identification via phylogenetic inference. *Nat Commun* **9**, 5144 (2018). https://doi.org/10.1038/s41467-018-07627-7.

[2] Felsenstein, J. *Inferring Phylogenies* (Sinauer Associates, Sunderland, Massachusetts, 2004).

[3] Cingolani, P. *et al.* A program for annotating and predicting the effects of single nucleotide polymorphisms, snpeff: Snps in the genome of drosophila melanogaster strain w1118; iso-2; iso-3. *Fly* **6**, 80–92 (2012). https://doi.org/10.4161/fly.19695.

[4] Hoey, J. The Two-Way Likelihood Ratio (G) Test and Comparison to Two-Way Chi Squared Test. *arXiv* **1206.4881**, (2012). https://doi.org/10.48550/arXiv.1206.4881.
