# Supplementary material for: Phylogenetic inference reveals clonal heterogeneity in circulating tumor cell clusters
Source: Nat Genet. 2025 Jun 2;57(6):1357–61. doi: 10.1038/s41588-025-02205-2 (PMC12165839; doi:10.1038/s41588-025-02205-2)
Supplement: Supplementary file 2 — Reporting Summary [file 41588_2025_2205_MOESM2_ESM.pdf]

Corresponding author(s): Prof. Dr. Nicola Aceto

Last updated by author(s): Apr 2, 2025

Reporting Summary

Nature Portfolio wishes to improve the reproducibility of the work that we publish. This form provides structure for consistency and transparency in reporting. For further information on Nature Portfolio policies, see our Editorial Policies and the Editorial Policy Checklist.

Statistics

For all statistical analyses, confirm that the following items are present in the figure legend, table legend, main text, or Methods section.

n/a	Confirmed	
☐	☒	The exact sample size (*n*) for each experimental group/condition, given as a discrete number and unit of measurement
☒	☐	A statement on whether measurements were taken from distinct samples or whether the same sample was measured repeatedly
☐	☒	The statistical test(s) used AND whether they are one- or two-sided<br>*Only common tests should be described solely by name; describe more complex techniques in the Methods section.*
☒	☐	A description of all covariates tested
☒	☐	A description of any assumptions or corrections, such as tests of normality and adjustment for multiple comparisons
☐	☒	A full description of the statistical parameters including central tendency (e.g. means) or other basic estimates (e.g. regression coefficient) AND variation (e.g. standard deviation) or associated estimates of uncertainty (e.g. confidence intervals)
☐	☒	For null hypothesis testing, the test statistic (e.g. *F*, *t*, *r*) with confidence intervals, effect sizes, degrees of freedom and *P* value noted<br>*Give P values as exact values whenever suitable.*
☐	☒	For Bayesian analysis, information on the choice of priors and Markov chain Monte Carlo settings
☒	☐	For hierarchical and complex designs, identification of the appropriate level for tests and full reporting of outcomes
☐	☒	Estimates of effect sizes (e.g. Cohen's *d*, Pearson's *r*), indicating how they were calculated

Our web collection on statistics for biologists contains articles on many of the points above.

Software and code

Policy information about availability of computer code

Data collection

For exome sequencing analysis, paired-end reads were aligned to the GRCh38 human reference using BWA-mem algorithm (v0.7.15) and sorted using SAMtools (v.1.7). Xenograft samples were additionally aligned to the GRCm38 mouse reference genome and assigned to either human or mouse using Disambiguate (v1.0.0). Reads identified as mouse were removed from subsequent analysis. Deduplication of reads was performed on a per-sample basis using Picard MarkDuplicates (v.2.9.2) and local realignment was performed using the Genome Analysis Toolkit (GATK) IndelRealigner (v.3.7.0) at the sample and donor level to improve alignment accuracy around indels. Quality control, as well as coverage and exome enrichment statistics were generated using FastQC (v.0.11.8), CollectHsMetrics from Picard suite (v.2.9.0), and QualiMap (v.2.2.1) and visualized using MultiQC (v.0.8). Mpileup files were generated with SAMtools (parameters: -q 40 -Q 30) at donor level and variants were called using SCIΦ on all samples from the same donor simultaneously. The variant annotation and effect prediction tool SnpEff (v.5.2a) was used to classify observed genetic variants by putative impact on protein functionality, using default parameters and variant calling format (VCF) files as input. The Cancer Genome Interpreter (CGI) web tool was used to analyse genetic variants by their predicted oncogenic capacity. For the barcoding analysis, reads in FASTQ files were aligned to barcode reference sequences using bowtie2 (v2.5.1). Resulting SAM files were sorted using Samtools sort (v1.16.1) and the number of read segments mapped to each barcode reference sequence was counted using Samtools idxstats (v1.16.1). Resulting barcode count files were processed in R (v4.2.3, R Foundation for Statistical Computing) for secondary analyses. Original code to reproduce the phylogenetic analysis, as well as the analysis of barcoded xenograft samples, have been deposited to GitHub (https://github.com/cbg-ethz/CTC-SCITE) under the GPL-3.0 license and archived at Zenodo (10.5281/zenodo.12774098).

Data analysis	Statistical testing and visualizations were conducted in R (v4.2.3, R Foundation for Statistical Computing). Graphical Illustrations were generated using BioRender and Adobe Illustrator (v28.6). Microscopic images were processed using the Fiji image processing software (v2.14.0). Phylogenetic inference was conducted using a custom software implemented in C++ and publicly available through https://github.com/cbg-ethz/CTC-SCITE under the GNU General Public License v3.0.

For manuscripts utilizing custom algorithms or software that are central to the research but not yet described in published literature, software must be made available to editors and reviewers. We strongly encourage code deposition in a community repository (e.g. GitHub). See the Nature Portfolio guidelines for submitting code & software for further information.

Data

Policy information about availability of data

All manuscripts must include a data availability statement. This statement should provide the following information, where applicable:

- Accession codes, unique identifiers, or web links for publicly available datasets
- A description of any restrictions on data availability
- For clinical datasets or third party data, please ensure that the statement adheres to our policy

The sequencing datasets that support the findings of this study have been deposited in the European Nucleotide Archive (ENA, EMBL-EBI; accession number PRJEB77733). The sequencing datasets for samples initially included in Szczerba et al. (Nature, 2019) are deposited under ENA accession number PRJEB24623 (Supplementary Table 3). The genome references used in this study were obtained from Gencode (https://www.gencodegenes.org/human/release_32.html for GRCh38 and https://www.gencodegenes.org/mouse/release_M24.html for GRCm38).

Research involving human participants, their data, or biological material

Policy information about studies with human participants or human data. See also policy information about sex, gender (identity/presentation), and sexual orientation and race, ethnicity and racism.

Reporting on sex and gender	Both male and female patients were included in the study. We consider our findings to be independent of patient sex or gender, thus sex and gender were not considered in our study design and no sex- or gender-based analyses were performed.
Reporting on race, ethnicity, or other socially relevant groupings	Patients were not cohorted and our study does not include any comparisons between individual cohorts. Thus, we do not consider race, ethnicity or other socially relevant grouping relevant for this study.
Population characteristics	Our patient cohort consisted of seven female patients with breast cancer as well as two male patients with prostate cancer (Supplementary Table 1, sex based on self-reporting). We consider our findings to be independent of patient sex or gender, thus sex and gender were not considered in our study design and no sex- or gender-based analyses were performed.
Recruitment	Clinicians recruited the patients by detailed explanation of the project workflow, risks, patients' rights and how the donated samples were encrypted. No specific bias in recruitment was identified. Patients were informed about the impact that our study could have on future cancer research. Clinicians replied to all the questions that patients raised. Patients were given the time to think and decide in free will. The patients did not receive any participant compensation.
Ethics oversight	All specimens were obtained at the University Hospital Basel under the study protocols EKNZ BASEC 2016-00067, EKNZ 2014-329 and EK 321/10, approved by the Swiss authorities (EKNZ, Ethics Committee northwest/central Switzerland) and in compliance with the Declaration of Helsinki.

Note that full information on the approval of the study protocol must also be provided in the manuscript.

Field-specific reporting

Please select the one below that is the best fit for your research. If you are not sure, read the appropriate sections before making your selection.

☒ Life sciences ☐ Behavioural & social sciences ☐ Ecological, evolutionary & environmental sciences

For a reference copy of the document with all sections, see nature.com/documents/nr-reporting-summary-flat.pdf

Life sciences study design

All studies must disclose on these points even when the disclosure is negative.

Sample size	Animal study design: sample sizes were determined while adhering to 3R (replace, reduce, refine) principles based on our previous experience (Diamantopoulou, Z. et al. The metastatic spread of breast cancer accelerates during sleep. Nature 607, 156–162 (2022); Szczerba, B. M. et al. Neutrophils escort circulating tumour cells to enable cell cycle progression. Nature 566, (2019)) and without predetermined calculations.
Data exclusions	No data was excluded from the studies.
Replication	Evidence for the presence of oligoclonal CTC clusters was confirmed in multiple patients (n = 9) with different cancer types. 426 CTC clusters from total 12 animals were used to infer an association of CTC cluster clonality with primary tumor diversity and CTC cluster size. This association was confirmed in two independent experiments (n=7 and n=5), demonstrating reproducibility of our findings.

Randomization	All mice were randomized before mouse experiments and blindly selected before tumor cell injection. Group allocation was predetermined by the number of cells injected.
Blinding	Patient samples were encrypted. For the barcoding analysis, encrypting animals was not appropriate due to obvious differences in tumor growth dynamics between experimental categories.

Reporting for specific materials, systems and methods

We require information from authors about some types of materials, experimental systems and methods used in many studies. Here, indicate whether each material, system or method listed is relevant to your study. If you are not sure if a list item applies to your research, read the appropriate section before selecting a response.

Materials & experimental systems

n/a	Involved in the study
☐	☒ Antibodies
☐	☒ Eukaryotic cell lines
☒	☐ Palaeontology and archaeology
☐	☒ Animals and other organisms
☒	☐ Clinical data
☒	☐ Dual use research of concern
☒	☐ Plants

Methods

n/a	Involved in the study
☒	☐ ChIP-seq
☒	☐ Flow cytometry
☒	☐ MRI-based neuroimaging

Antibodies

Antibodies used	EpCAM-AF488 (Cell Signaling Technology, CST5198, clone VU1D9, 1:50), HER2-AF488 (BioLegend, 324410, clone 24D2,1:50), EGFR-FITC (GeneTex, GTX11400, clone ICR10, 1:25), and CD45-BV605 (BioLegend, 304042, clone HI30, 1:25) antibodies were used in this study.
Validation	Antibody sensitivities and specificities were confirmed through successful application in a variety of studies leading to peer-reviewed publications in top-tier journals, e.g. "Diamantopoulou Z. et al. 2022, Nature" for HER2-AF488, CD45-BV605 and EGFR-FITC antibodies and "Manuel C Scheidmann, et. al. 2022, Cancer Research" for the EpCAM-AF488 antibody.

Eukaryotic cell lines

Policy information about cell lines and Sex and Gender in Research

Cell line source(s)	Human CTC-derived BR16 cells were generated from a female patient with hormone receptor-positive breast cancer at the University Hospital Basel. MDA-MB-231 LM2 human breast cancer cells (female origin) were obtained from J. Massagué, Memorial Sloan Kettering Cancer Center.
Authentication	The cell lines were not authenticated. Authentication is not applicable for the human CTC-derived BR16 cells and the MDA-MB-231 LM2 human breast cancer cells.
Mycoplasma contamination	All cell lines were tested negative for mycoplasma.
Commonly misidentified lines (See ICLAC register)	No misindentified lines were used in this study.

Animals and other research organisms

Policy information about studies involving animals; ARRIVE guidelines recommended for reporting animal research, and Sex and Gender in Research

Laboratory animals	The study involved 8-10 weeks-old female NOD.Cg-Prkdcscid-Il2rgtm1Wjl/SzJ (NSG) mice.
Wild animals	This study did not involve wild animals.
Reporting on sex	All animals included in this study were female in order to match the sex of the donors of the engrafted human breast cancer cells.
Field-collected samples	This study did not involve samples collected from the field.
Ethics oversight	All mouse experiments were carried out according to institutional and cantonal guidelines (mouse protocol number 33688, approved by the cantonal veterinary office of Zurich).

Note that full information on the approval of the study protocol must also be provided in the manuscript.

Plants

Seed stocks	NA
Novel plant genotypes	NA
Authentication	NA
